# Laparoscopic Appendectomy in Children: Preliminary Study in Pediatric Hospital Albert Royer, Dakar

**DOI:** 10.1155/2015/878372

**Published:** 2015-09-10

**Authors:** Mbaye Fall, Doudou Gueye, Ibrahima Bocar Wellé, Faty Balla Lo, Aloise Sagna, Marie Diop, Ibrahima Fall

**Affiliations:** Pediatric Surgery Department, Children Hospital Albert Royer, Dakar, Senegal

## Abstract

Appendiceal pathology's management has benefited in recent years from the advent of laparoscopic surgery. This study is to make a preliminary assessment of laparoscopic management of acute and complicated appendicitis in children after a few months of practice at the University Hospital Albert Royer, Dakar. This is a retrospective study of 22 cases of patients, all operated on by the same surgeon. The parameters studied were age, sex, clinical data and laboratory features, radiological data, and results of surgical treatment. The mean age of patients was 9.5 years with a male predominance. The series includes 14 cases of acute appendicitis and 8 complicated cases. Appendectomy anterograde is practiced in 81% of cases. Appendectomy was associated with peritoneal wash in 17 patients including 9 cases of acute appendicitis. Drainage of Douglas pouch is performed in 2 patients with complicated appendicitis; the average production was 300 cc of turbid liquids and any complications were not founded. An abscess of Douglas pouch is noted in 2 patients with complicated appendicitis undrained. These Douglas abscesses were treated medically. No conversion of laparotomy was performed in the series. After an average of 8 months no other problems were noted.

## 1. Introduction

Appendiceal pathology is 15% to 20% of abdominal surgical emergencies in pediatrics [1, 2]. Its management has benefited in recent years with the advent of laparoscopic surgery. Many authors recognize indeed the benefits of this surgical method and many teams practice it around the world [3–5]. Appendiceal pathology is the primary indication for laparoscopy in pediatric patients [6, 7]. In our context laparoscopic appendectomies are performed in adult surgery since 2000 with good results [8]. Our study aims to make a preliminary assessment of laparoscopic management of acute and complicated appendicitis in children after a few months of practice.

## 2. Material and Method

**Figure 1:**
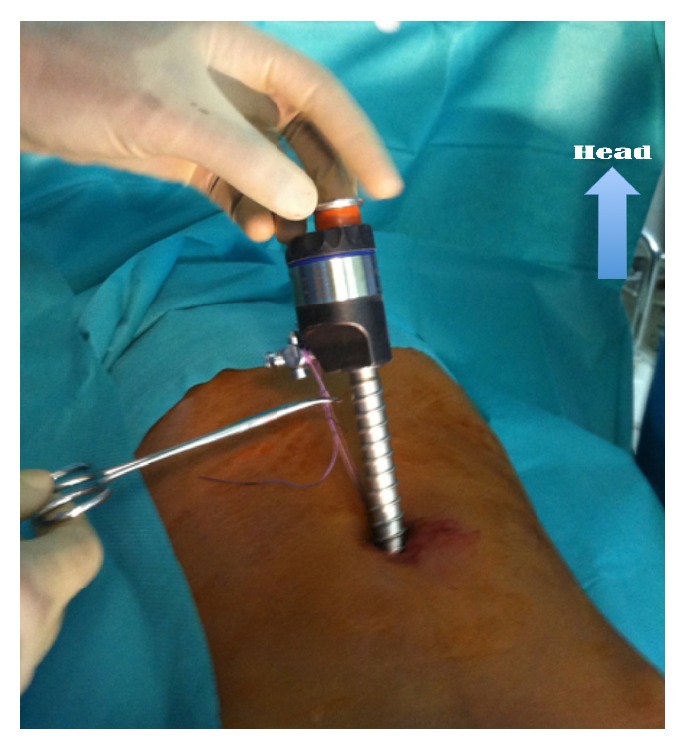
Open coelioscopy (umbilical trocar fixation during laparoscopic appendectomy).

**Figure 2:**
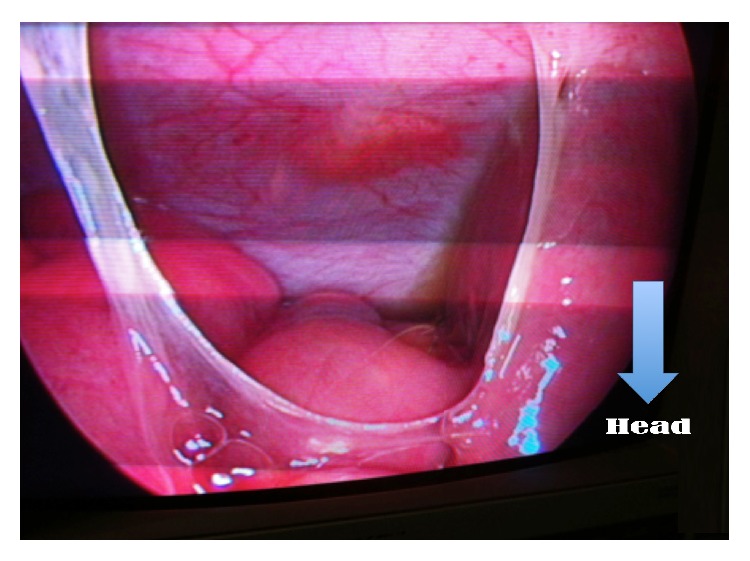
Laparoscopic view of appendicular appendicitis: adhesions and turbid liquids in peritoneal cavity.

**Figure 3:**
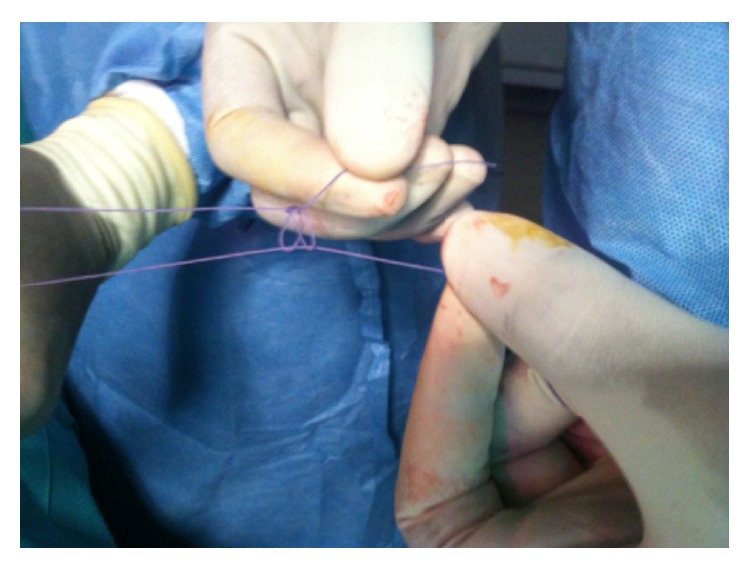
Extracorporeal knot used for ligation of the base of the appendix.

**Figure 4:**
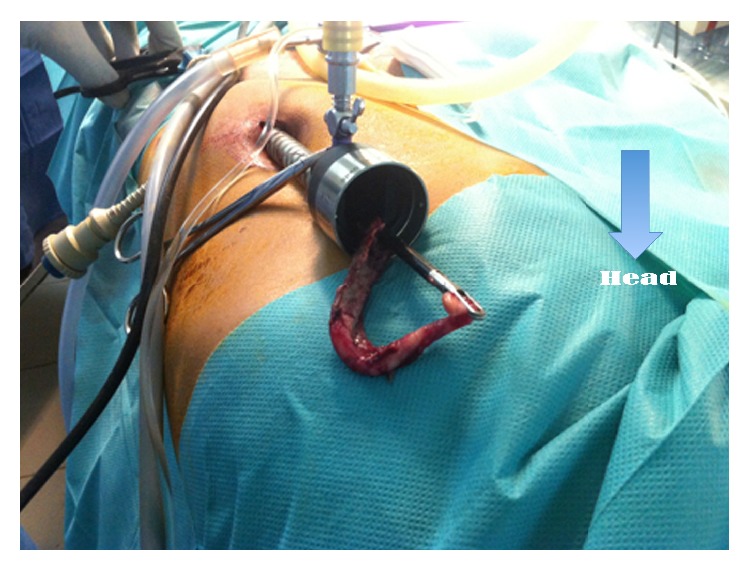
Extraction of the appendix through the umbilical trocar.

**Figure 5:**
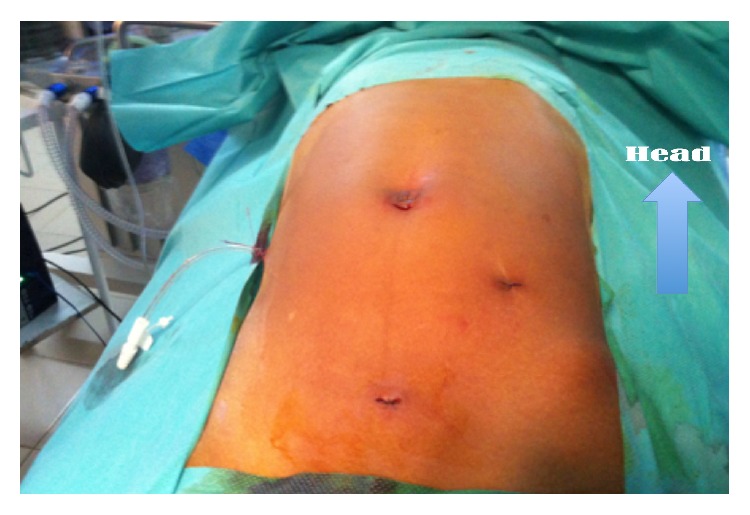
Appendicular peritonitis operated laparoscopically and drained.

This is a retrospective study about patients incurring laparoscopic appendectomy in the period from May 2013 to November 2014 in the Pediatric Surgery Department, Hospital Albert Royer, Dakar. We used general anesthesia with orotracheal intubation, nasogastric tube, and a urinary catheter. The patient is supine with the left arm along the body. The operator and his assistant are placed at the left of the patient and column laparoscopy is facing surgeons. The umbilicus is gripped between two Kelly clamps, everted, and opened. The fascial hole is then expanded and the peritoneum opened with a pair of scissors. We introduce an umbilical trocar around the fascia hole. The “open coelioscopy” finishing the wire is tight and rolled at the end of the umbilical trocar and then clamped by a Kelly's clamp (Figure 1). The CO_2_ insufflation is started at a pressure of 12 mmHg and a flow rate of 4 L/minute. Two 5 mm trocars are then placed a finger's breadth above the pubic bone and on the left iliac fossa. A scanning optics and a sequence of intestines allow exploration of the abdominal cavity (Figure 2). Hemostasis is made by coagulating the mesoappendix sometimes with bipolar hook. For ligation of the appendicular base we use a lasso of resorbable wire 3/0 handmade in extracorporeal and introduced by 5 mm trocar the left iliac fossa (Figure 3). The lasso is threaded around the appendix and is tight at the base. The appendix section is made above the node and the appendix is immediately extracted through the umbilical trocar (Figure 4). Depending on the case a suction-washing is optionally performed with a drainage (Figure 5). The trocars are removed under direct vision followed by a full exsufflation pneumoperitoneum. Umbilical foramen is tight in order to close the opening fascia. Trocar orifices of 5 mm are closed by 1 or 2 points of resorbable wire 4/O. A bandage is placed on the umbilicus for a period of 5 days.

All patients were operated on by the same surgeon. The parameters studied were age, sex, clinical data, laboratory features, radiological data, and results of surgical treatment.

## 3. Results

We realized 22 appendectomies by laparoscopy like 14 boys and 8 girls against 36 appendectomies open surgery, like 38%. The mean age of patients was 9.5 years (7 years and 15 years). Appendectomies were performed emergently except in one case. The patients were examined after an average of 2 days (1 to 10 days) with abdominal pain and vomiting as predominant symptomatology. The abdominal radiography realized in 8 cases showed an appendicolith in one case. An abdominal ultrasound performed in 18 patients confirmed the diagnosis of appendiceal pathology with increasing size of the appendix and infiltration of fat perished appendix, usually associated with peritoneal effusion. Laboratory tests included a blood count, blood grouping, and prothrombin time. No patient had a C-reactive protein count. The diagnosis before surgical exploration revealed 16 cases of acute appendicitis, 3 abscesses, 2 peritonitis, and 1 appendicular lump. Appendectomy was postponed for one month after antibiotic therapy with ciprofloxacin and metronidazole in appendicular lump. Surgical exploration was performed after 1 or 2 days (2 and 4 days) and corrected preoperative diagnosis in 3 cases. Then an operative discovery of 1 appendicular lump and 2 appendicular peritonitides was performed in patients initially taken, respectively, appendiceal abscess and acute appendicitis.

We realized 18 anterograde appendectomies and 4 retrograde appendectomies. Appendectomy was associated with a suction-washing in 9 cases of acute appendicitis, 2 cases of appendicular abscess, 4 cases of peritonitis, and 2 cases of appendicular lump. The establishment of a drain after peritoneal lavage was necessary in patients with appendiceal abscess and 2 cases of peritonitis. The drain was removed on the fifth postoperative day. The mean operating time was 70 min (35 min–120 min). No conversion to laparotomy was performed. Feeding was allowed one day after surgery for acute appendicitis while for complicated forms it had begun the second or third postoperative day. The majority of patients was discharged on the first postoperative day. Operative complications were food vomiting in 3 patients, scapular pain in 2 patients, and abscess Douglas in 2 patients (Table 1). No parietal suppuration was noted. The average hospital stay was 5 days (2 and 11 days). After a mean of 8 months no other problems were noted. In the series there is no mortality.

## 4. Discussion

Laparoscopy in pediatric surgical is practiced in our country since 20 months. Appendectomy is the first indication followed by cholecystectomy; the reason could be the high incidence of appendiceal pathology but also the simplicity of the surgical gesture and then constituting an excellent tool apprenticeship to laparoscopic surgery [3, 9]. The acute appendicitis is a childhood disease, more common in males [10–16]. These results are the same with ours that found an average age of 9.5 years, with 63% of males. In our study the indications of laparoscopy were more inclined towards acute appendicitis which constitutes 63% of the series, in our context there are more complicated cases [17]. The high frequency of complicated appendicitis is secondary to long diagnostic delay. As the delay is long, the risk of complications is important [18]. In most cases the reasons of these complications are long delay, traditional medication, and inappropriate use of antibiotics [17, 19]. Some authors estimate that 91% of acute appendicitises are seen before 3 days [18]. In our series we had more acute cases with an average consultation within 2 days. Long delays concern complicated shapes. These data are illustrated by a study including all appendectomies, like a delay of 6 days and 74% of complicated appendicitis [17]. Clinical examination should be coupled with ultrasound when there is a doubt in diagnosis [20]. Sometimes ultrasound is responsible for false positive or false negative or even mistaking the anatomical shape [21]. She was performed in 18 of our patients with 3 misdiagnoses, adjusted during the surgical exploration. Ultrasound nevertheless remains a gold standard for appendiceal disease [22–25].

However, in our current working conditions, laparoscopy is still debated in complicated appendicitis because of the risk of morbidity like septic shock or gas embolism by expanded vessels consequence of CO_2_ insufflation peritoneum [26], a lack of standardized antibiotic protocol governing the surgical procedure and socioeconomic problems. For these reasons some pediatric surgeons still prefer open surgery associated with drainage of the peritoneal cavity for complicated appendicitis [17]. In our series preoperative intravenous antibiotic therapy was recommended in 40.6% of cases based on beta-lactam, aminoglycoside, and nitroimidazole. Postoperative antibiotic therapy was initially administered parenterally and on orally relay, with an average of 7.5 days of administration. Many combinations have been proposed in pediatrics. Several authors argue that antibiotic therapy is especially necessary in complicated appendicitis, and the treatment is started intraoperatively always pursuing parenteral postoperatively, with adaptation of the antibiogram, and relays orally after 48 hours of apyrexia [18, 27].

Laparoscopy is more controversial in appendicular lump [9]. In our series we had 8 complicated shapes including 3 cases peroperatively discovered and a case of appendicular lump operated on after a month of antibiotics. These complicated appendicitises have all benefited from an appendectomy and peritoneal toilet. The 2 patients who underwent drainage of the peritoneal cavity showed an uneventful postoperative course with an average production of 300 cc of turbid liquids. However we have 40% morbidity in patients with complicated appendicitis operated on urgently and undrained. These patients presented a Douglas abscess that was treated with antibiotic therapy and rehabilitation puncture by endoanal track. These data raise the interest of the Douglas drain in complicated appendicitis already advocated by some authors [5, 28]. We should probably need a large-randomized study to hope to have recommendations on the usefulness of laparoscopic drainage of complicated appendicitis in our context. This complication has been reported by some authors [16, 29]. Medical treatment appears effective in the absence of bowel obstruction [30].

The mean operating time during the study was 70 minutes conformable to that of the literature [8, 31].

Laparotomy conversion varies between 0 and 11% with an average rate of 2.8%. The conversion to laparotomy is performed for appendiceal chest for an ectopic position of the appendix for a ruptured appendix or a Meckel's diverticulum [29]. In our series we have not made any conversion to laparotomy. Other conversion reasons such as coagulation disorders and technical difficulties are reported by other authors [32–34].

Laparoscopy provides the surgeon working comfort and precision answering the first basic rules of surgery “to see in order to properly operate” [35]. It is no longer considered a luxury but an important surgical breakthrough as it can resolve many problems encountered in open surgery. The promotion of laparoscopy in our country requires an extension of the training centers, strengthening of the technical facilities, and the creation of learned society.

## 5. Conclusion

Laparoscopy is an appropriate and effective surgical approach especially in acute appendicitis. However, for complicated appendicitis its usefulness is still debated in our context. Drainage of Douglas' pouch could be a valuable aid for the reduction of residual intraperitoneal collections in the management of complicated forms by laparoscopy.

## Figures and Tables

**Table 1:** The patients' characteristics.

	Acute ap.	Abscess	Péritonitis	Ap. lump
Sex (M/F)	9/5	2/0	2/2	1/1
Mean age (yr)	7	10	11	9,5
Appendectomy (A/R)	14/0	1/1	2/2	1/1
Suction-washing	9	2	4	2
Drainage	0	1	2	0
Operative time (min)	55 (35–72)	62 and 84	74 (65–120)	58 and 104
Conversion	0	0	0	0
Complications	2 scapular pain, 1 vomiting	1 Douglas abscess, 1 vomiting	1 Douglas abscess	1 vomiting
Hospital stay (days)	4 (2–6)	5 and 12	7 (5–14)	2 and 7

A = antérograde, R = rétrograde, M = male, F = female, acute ap. = acute appendectomy, and ap. lump = appendicular lump.
